# Challenges and Opportunities for Celecoxib Repurposing

**DOI:** 10.1007/s11095-023-03571-4

**Published:** 2023-08-08

**Authors:** Urszula Bąk, Anna Krupa

**Affiliations:** Department of Pharmaceutical Technology and Biopharmaceutics, Faculty of Pharmacy, Jagiellonian University Medical College, 9 Medyczna Street, 30-688 Cracow, Poland

**Keywords:** Coxibs, Metronomic therapy, Hand-foot syndrome, Mental disorders therapy, Antimicrobial activity, Long-acting injectables, Mesoporous carriers, Spanlastics

## Abstract

Drug repositioning, also known as drug repurposing, reprofiling, or rediscovery, is considered to be one of the most promising strategies to accelerate the development of new original drug products. Multiple examples of successful rediscovery or therapeutic switching of old molecules that did not show clinical benefits or safety in initial trials encourage the following of the discovery of new therapeutic pathways for them. This review summarizes the efforts that have been made, mostly over the last decade, to identify new therapeutic targets for celecoxib. To achieve this goal, records gathered in MEDLINE PubMed and Scopus databases along with the registry of clinical trials by the US National Library of Medicine at the U.S. National Institutes of Health were explored. Since celecoxib is a non-steroidal anti-inflammatory drug that represents the class of selective COX-2 inhibitors (coxibs), its clinical potential in metronomic cancer therapy, the treatment of mental disorders, or infectious diseases has been discussed. In the end, the perspective of a formulator, facing various challenges related to unfavorable physicochemical properties of celecoxib upon the development of new oral dosage forms, long-acting injectables, and topical formulations, including the latest trends in the pharmaceutical technology, such as the application of mesoporous carriers, biodegradable microparticles, lipid-based nanosystems, or spanlastics, was presented.

## Introduction

**Fig. 1:**
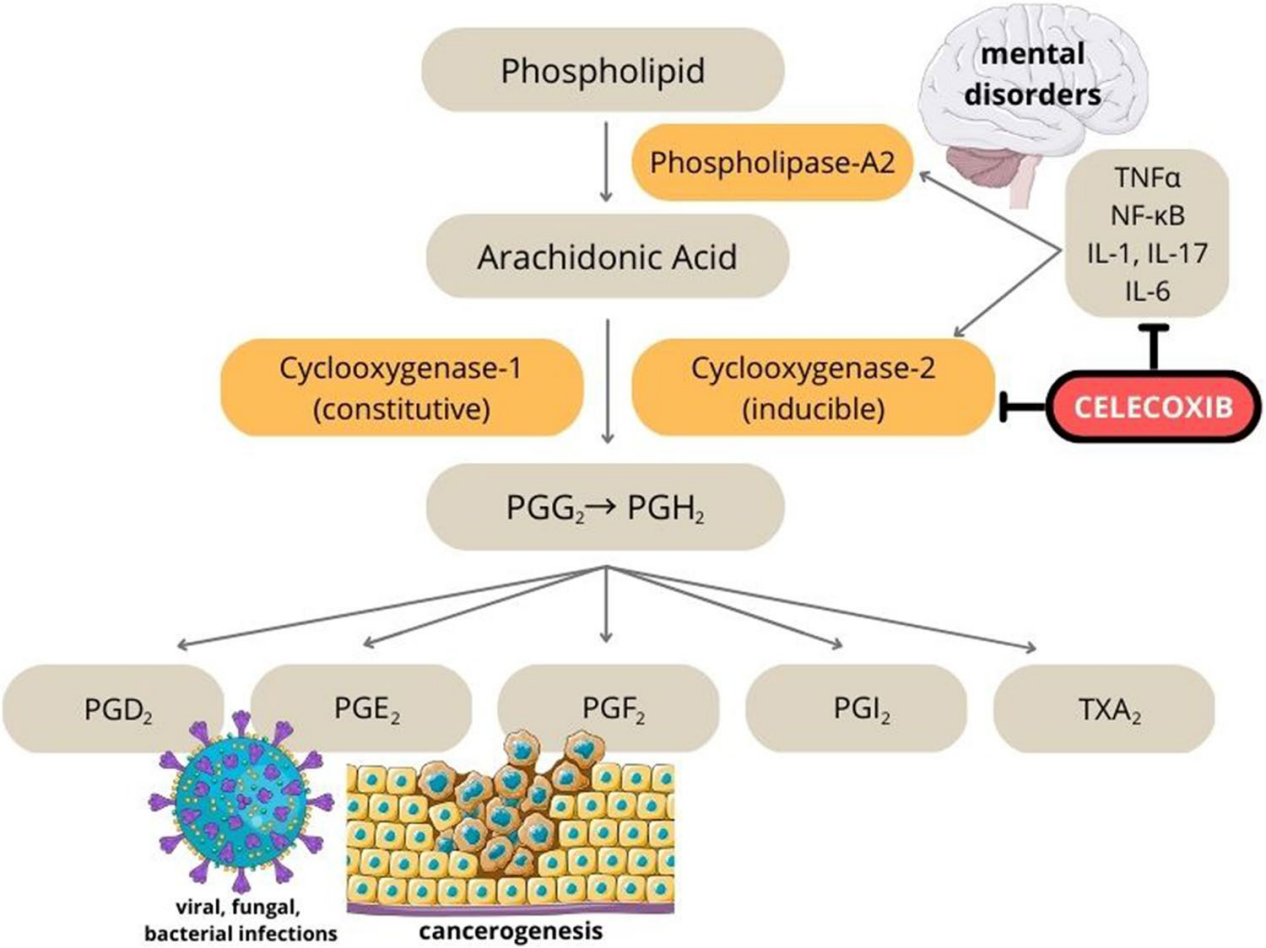
Arachidonic acid cascade and effects of celecoxib on COX-2 related biochemical pathways

Celecoxib is a non-steroidal anti-inflammatory drug (NSAID) that represents the class of selective COX-2 inhibitors (coxibs) that block cyclooxygenase-2 (COX-2), an enzyme that participates in the conversion of arachidonic acid to prostaglandins [1]. The scheme of that conversion is shown in Fig. 1. Importantly, under physiological conditions, COX-2 is not detected in human tissues and its expression is triggered by inflammation. Therefore, inhibition of COX-2 process is responsible for the anti-inflammatory, antipyretic, and analgesic properties of the drug [2].

From a chemical point of view, celecoxib is a pyrazole compound, a benzenesulfonamide of 4-[5-(4-methylphenyl)-3-trifluoromethyl-1*H*-pyrazol-1-yl]. COX-2 selectivity, anti-inflammatory activity, and *in vivo* efficacy are defined by the presence of a sulfonamide moiety in its structure [3]. A detailed crystallographic analysis of this compound carried out by Wang *et al.* [4] revealed that the 4-methyl phenyl and trifluoromethyl-1*H*-pyrazol rings are perpendicular to the benzenesulfonamide ring. Moreover, celecoxib forms dimers stabilized by N—H···O, C—H···N or C—H···π hydrogen bonds. Importantly, this single-component crystalline compound shows a unique mechanical behavior, that is, elastic flexibility and high stiffness related to aromatic C—H bonds.

There are three polymorphic forms of celecoxib, that is, form I, form II, and form III. Their melting points are 162.8 °C, 161.5 °C and 160.8 °C respectively [5]. Among them, form III is the most thermodynamically stable under ambient conditions. Celecoxib belongs to class II of the Biopharmaceutical Classification System (BCS) as a drug that is poorly soluble in water (< 5 µg/mL at 5–40 °C) and highly permeable [5, 6]. According to Lipinski’s rule of 5 in order to ensure intestinal absorption, the value of LogP should not be higher than 4, but for drugs administered orally, it should preferably be within the range of 1.38–1.80. Celecoxib is a highly lipophilic drug (LogP = 3.5) that shows weakly acidic properties related to the ionization of the sulfonamide group with a pKa value of 11.1 [5]. However, in contrast to other nonsteroidal anti-inflammatory drugs, there is no truly acidic proton in its structure. Thus, the solubility of celecoxib does not depend on the pH if studied within the physiological range.

According to data published in the New Drug Application (NDA) no 20–998 in 1998, after oral administration of Celebrex® capsules, the relative bioavailability of the drug in humans was 99%. However, it should be stressed that, because of the poor solubility of celecoxib, its absolute bioavailability in humans has not been determined. After oral administration of Celebrex®, its maximum plasma levels occurred after approximately 3 h, but the limited solubility of the drug resulted in high variability in absorption and prolonged elimination (t_0.5_ = 11–15 h). Celecoxib bonds strongly to proteins, mainly albumin (approx. 97%). It is metabolized by liver and its cytochrome enzymes: CYP2C9 and CYP3A4. Although celecoxib is not metabolized by CYP2D6, the drug binds to this cytochrome, inhibiting it. The liver metabolites of celecoxib are inactive derivatives of hydroxy, carboxy, and glucuronide that are eliminated by urine and feces [7].

### Authorized Single Drug Preparations

In December 1998, the FDA approved the celecoxib drug for the symptomatic treatment of osteoarthritis and adult rheumatoid arthritis. Celecoxib was found to be as effective a painkiller as other NSAIDs, i.e., diclofenac and ibuprofen, but importantly, this therapy was associated with fewer gastrointestinal side effects compared to previously mentioned NSAIDs [8]. Meanwhile, elevated levels of COX-2 were found in many premalignant lesions and epithelial cancers. It was assumed that blocking prostaglandin synthesis by inhibition of COX-2 may induce the cell death and be responsible for anticancer effects. As a result, in October 2003, the European Medicines Agency (EMA) granted an orphan designation to celecoxib (Pfizer Onsenal capsules) which started its clinical evaluation in the prevention of a rare disease, that is, familial adenomatous polyposis (FAP) as an adjunct to surgery and further endoscopic surveillance. In this rare disease, patients develop multiple polyps in the colon and rectum before the age of thirty, which strongly predispose them to colorectal cancer in the fourth or fifth decade of life. To prevent it, 400 mg of celecoxib was administered twice daily. However, due to the slow enrollment in this clinical trial and insufficient data to prove that the benefits of the drug outweigh risks, the marketing authorization for Onsenal was voluntarily withdrawn by Pfizer in March 2011.

Shortly after the orphan designation was granted to celecoxib, severe cardiovascular adverse effects for other coxib, i.e. rofecoxib (Vioxx by Merck Sharp & Dohme) were reported. Since then, celecoxib has been contraindicated in Europe in patients with known cardiovascular disease [9]. Currently, the daily dose of celecoxib considered safe is 200 mg per day [1]. Commercially, celecoxib is available in the form of oral capsules (50–400 mg).

In 2020 celecoxib was also registered in the US in the form of a single dose oral solution Elyxyb™ (120 mg/4.8 mL). In fact, it was a self-microemulsifying drug delivery system (SMEDDS) composed of 49.5% of lipids (PEG-8 caprylic/capric glycerides), 40.5% of surfactants (Tween 20 and propylene glycol monocaprylic ester [3:1]) and 10% of celecoxib [10]. This formulation allowed to improve the pharmacokinetic profile of the drug after its oral administration as compared to capsules [11]. In 2021, this product has been approved by the American Headache Society for the acute migraine treatment in adults. Interestingly, it was the first successful SMEDDS application for this clinical indication [10].

### Authorized Fixed-Dose Combination Drugs Loaded with Celecoxib

In May 2018, the FDA approved the first fixed-dose combination drug (FDCD) in the form of tablets destined for the therapy of coexisting hypertension and osteoarthritis. This formulation was launched by Purple Biotech, Ltd. (previously Kitov Pharmaceuticals Ltd., Israel) under the commercial name Consensi®. The drug product is composed of celecoxib and amlodipine besylate (a calcium channel blocker). It is available in three fixed dose combinations to be taken once a day. The dose of celecoxib is kept at 200 mg, while the dose of amlodipine varies from 2.5 mg to 10 mg. Since celecoxib can cause variations in blood pressure [12], a clinical trial was carried out to investigate the efficacy of this drug combination compared to monotherapy and *placebo* in a 2-week therapy (NCT02172040). The patients were treated with tablets loaded with the highest dose of amlodipine, which was 10 mg, while the dose of celecoxib was kept at 200 mg. The efficacy of both treatment schemes was similar and there were no differences in adverse effects between these groups. However, the treatment time was short, so it was difficult to predict long-term tolerance to this combination therapy [12, 13].

Three years later, in October 2021, the FDA approved Seglentis® tablets (Esteve Pharmaceuticals S.A., Spain) loaded with 56 mg of celecoxib and 44 mg of *rac*-tramadol hydrochloride (an opioid agonist) coprocessed to co-crystals (100 mg per tablet). This drug was aimed at the therapy of acute pain in adults. The novelty of this formulation relied not only on the simple combination of these two drugs, but also on a modification of their intrinsic dissolution and pharmacokinetics by covalent bonds created between these two structures that link them in a 1:1 molar ratio [14, 15]. The clinical advantage of this cocrystal-based formulation is faster celecoxib release, which may accelerate its absorption and shorten the onset of analgesic effect, while the slow release rate of tramadol leads to a reduction of its maximum plasma concentration, adverse effects, and prolong its therapeutic action [14]. In fact, the results of the phase III clinical trial (NCT03108482) confirmed that oral administration of 200 mg of this cocrystal formulation twice a day was more efficient in postoperative pain control in patients who underwent buninectomy than monotherapy with tramadol (Ultram® 50 mg every 6 h) or celecoxib (Celebrex® 100 mg twice a day). Importantly, no serious adverse reactions were reported after 48 h of treatment.

Another interesting fixed-dose combination drug loaded with celecoxib and ciprofloxacin (a fluoroquinolone antibiotic) is PrimeC tablets developed by NeuroSense Therapeutics (Cambridge, MA, USA). This formulation has an orphan drug status both in the US and in the EU and is currently prepared for phase IIb/III clinical trials for inhibition of amyotrophic lateral sclerosis (ALS) progression. This disease affects motor neurons in the brain and spinal cord, which result in muscle paralysis and eventually death within a few years after symptoms onset. The pathophysiology of ALS is still unclear and appears to be multifactorial, including neuroinflammation, RNA dysfunction, and iron accumulation [16]. Since ciprofloxacin is known for its chelating properties and its ability to regulate RNA function, it was combined with the anti-inflammatory drug celecoxib. Preclinical studies carried out by Goldshtein *et al.* [17] in a zebrafish ALS model confirmed that such a combination improves locomotor and cellular deficits more than each of the drugs tested alone. The results of the phase IIa study were positive and provided evidence that PrimeC was effective, safe, and well tolerated [16]. According to the information published on the NeuroSense website in mid-November 2022, the patients are enrolled for the trial in Israel, and the patient recruitment in the EU has been planned.

## Literature Search Strategy

**Fig. 2:**
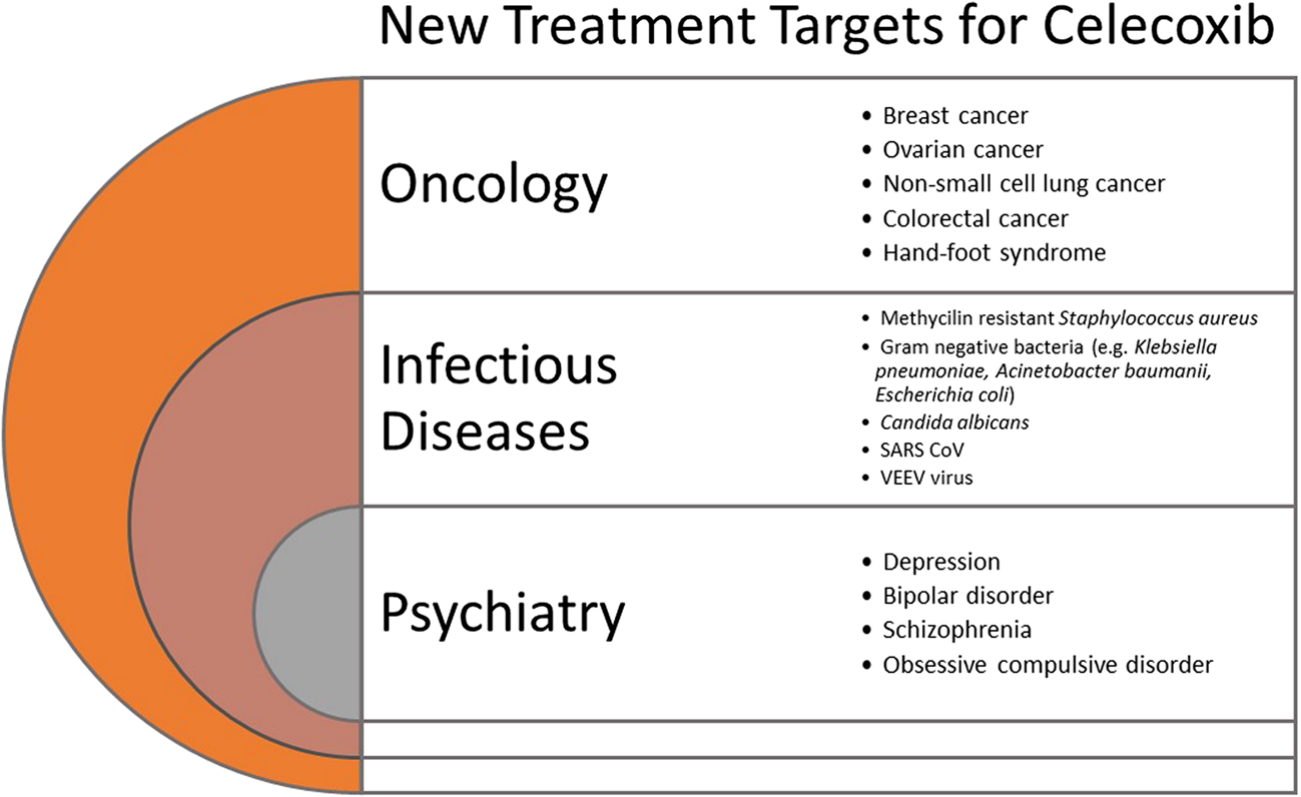
Potential new treatment targets for celecoxib

Since there has been growing interest in the search for new clinical indications for celecoxib, the present review aims to present new pathways in celecoxib repurposing. Databases such as MEDLINE PubMed, Scopus, and the registry of clinical trials (ClinicalTrials.gov) were explored by the US National Library of Medicine (NLM) at the US National Institutes of Health (NIH). The searches were carried out between September 7th 2022 and the 7^th^ of January 2023. The search strategy relied on the use of the basic keyword ‘celecoxib’ (e.g. > 7 800 citations in PubMed) followed by the advanced search ‘celecoxib’ AND (‘repurposing’ OR ‘repositioning’). When the records were generated, the search was restricted to the citations from the last decade, which, for example, resulted in more than 3800 and 40 citations found in PubMed for the basic and advanced search, respectively. Based on these outcomes, a list of new indications for celecoxib was created (Fig. 2). The final search was performed using the keyword ‘celecoxib’ AND ‘the name of the indication of interest’ or ‘celecoxib’ AND ‘route of administration’, but this time without the use of the citation date filter.

## Novel Treatment Targets

### Antineoplastic Activity and Cancer Therapy

Several reports have provided evidence that the overexpression of COX-2 is typical of neoplastic (premalignant and malignant) tissues supporting the theory of the inflammation-related pathway of carcinogenesis [18]. This overexpression is related to increased transcription and enhanced stability of mRNA. It should be noted that high levels of this enzyme were observed especially in poorly differentiated and deeply invasive cancer cells [19]. Furthermore, it was stated that NSAIDs are able to control inflammation in cancer cells by reducing the level of various signaling molecules that are responsible for tumor progression and metastasis, i.e. TNF-α (tumor necrosis factor α), NF-κB (nuclear factor kappaB) or VEGF (vascular endothelial growth factor) [20, 21].

In addition to these aforementioned mechanisms, celecoxib is also able to induce apoptosis by opening the mitochondrial membrane via the reactive oxygen species signaling pathway [1]. In consequence, this drug can be an interesting option for the treatment of advanced invasive cancer, as well as for chemoprevention (Table I). Multiple studies aimed to assess whether there was a synergistic effect between celecoxib and standard radio or chemotherapy [22]. In fact, celecoxib was found to elicit immune cell-regulated tumor cell lysis [23], apoptosis [24], inhibited the cell cycle [25] and angiogenesis [26], preventing tumor progression and metastasis. These findings led to progress studies on the clinical efficacy of celecoxib in metronomic schedules of chemotherapy, which, *inter alia* can be a part of neoadjuvant or palliative cancer treatment. These schemes refer to the therapeutic concept, according to which cancer is a chronic disease. This idea dates back to the year 2000 when research work of Browder *et al.* [27] and Klement *et al.* [28] shed new light on cancer therapy, and ever since, there have been some successful clinical translations, mainly in the field of the palliative treatment of solid tumors, e.g. breast cancer, lung cancer, but also in the management of cancers of hematological origin.

**Table I.** Clinical Studies Carried Out to Evaluate the Efficacy of Oral Celecoxib Administration in New Treatment Targets. Daily Doses of Celecoxib are Listed

Indication	Type of clinical study	Phase	Sample size	Intervention	Control	Results	Clinical trial number	References
Breast cancer	Non-randomized	II	15 pts	Cyclophosphamide 50 mg + CXB 400 mg	-	MCT with cyclophosphamide and CXB shows a therapeutic effect in advanced breast cancer, mild side effects	n.a	[29, 30]
Ovarian cancer	Prospective	n.a	45 pts	Carboplatin (i.v.) + CXB 400 mg	-	Carboplatin combined with CXB shows promising activity in ovarian cancer,	NCT0112435	[31]
Prostate cancer	Adaptive, multiarm, randomized	n.a	1.245 pts	Zolendronic acid 4 mg (i.v.) + CXB 800 mg + HT/RTH or CXB 800 mg + HT/RTH	Standard hormone therapy or RTH	No evidence for improved survival in prostate cancer	n.a	[32]
	Prospective	II	22 pts	Docetaxel 25 mg/m^3^ (i.v.) + zolendronic acid 4 mg (i.v.) + CXB 400 mg	-	Docetaxel, zolendronic acid and CXB combined did not improve survival	n.a	[33]
	Retrospective	n.a	49 pts	Cyclophosphamide 50 mg + Dexamethasone 1 mg + CXB 400 mg	-	MCT was safe, effective and well tolerated	n.a	[34]
Non-small cell lung cancer	Randomized, double-blinded, *placebo* controlled	III	319 pts	First-line chemotherapy (carboplatin + gemcitabine or carboplatin + vinorelbine doses n.a.) + CXB 800 mg	First-line chemotherapy (carboplatin + gemcitabine or carboplatin + vinorelbine, doses n.a.) + *placebo*	No significant difference in overall survival	n.a	[35]
	Randomized, double-blinded, *placebo* controlled	II	312 pts	Carboplatin (i.v.) + pemetrexed 500 mg/m^3^ + CXB 800 mg or carboplatin (i.v.) + gemcitabine 1,000 mg/m^3^ + CXB 800 mg	Carboplatin (i.v., AUC 6) + pemetrexed 500 mg/m^3^ + *placebo* or carboplatin (i.v., AUC 5.5) + gemcitabine 1000 mg/m^3^ + *placebo*	No significant differences between CXB-treated groups and the control	n.a	[36]
COVID-19	Randomized, double-blinded, *placebo* controlled	I	67 pts	CXB 400 mg or 200 mg + routine treatment	Routine treatment	No significant differences between control and treatment groups in terms of symptoms	IRCT20200907 048644N1	[37]
	prospective	n.a	44 pts	CXB 400 mg or 200 mg + routine treatment	Routine treatment	Reduction of the PGE2 levels and promoted recovery of ordinary and severe COVID-19 in CXB groups	n.a	[38]
OCD	Randomized, double-blinded, *placebo* controlled	II-III	60 pts	SSRI, dose n.a + CXB 400 mg	SSRI dose n.a + *placebo*	Adjuvant treatment with CXB can improve obsessive and compulsive symptoms	IRCT20151223 5280N21	[39]
	prospective, double-blinded	n.a	56 pts	fluoxetine 20 mg + CXB 400 mg	fluoxetine 20 mg + *placebo*		n.a	[40]
	Randomized, double-blind, *placebo* controlled, parallel-group	II-III	54 pts	fluvoxamine 100 mg, 200 mg, after 4 weeks + CXB 400 mg	fluvoxamine 100 mg daily, followed by 200 mg daily after 4 weeks + *placebo*		IRCT20131218 1556N56	[41]

*CXB* celecoxib, *HT *hormone therapy, *i.v. *intravenous, *OCD *obsessive–compulsive disorder, *MCT *metronomic chemotherapy, *n.a. *not available, *pts *patients, *RTH *radiotherapy, *SSRI *selective serotonin reuptake inhibitors

In metronomic chemotherapy low drug doses are administered to patients for a long period of time in contrast to conventional cytotoxic regimens in which drugs are administered in maximal tolerated doses for a short period of time [42]. However, these drug doses still have to be high enough to get the clinical response. Importantly, this strategy gives the opportunity to overcome drug resistance by activating the antitumor response, and to prolong remission while considerably reducing side effects compared to conventional chemotherapy. Furthermore, patients can often be chronically treated at home without the need for hospitalization, as some drugs are administered orally, which also guarantees high compliance [43].

The treatment of breast cancer is the field in which both the efficacy and safety of various drug combinations have been investigated on the metronomic schedule. The coadministration of 200–400 mg of celecoxib per day together with 50 mg of cyclophosphamide to patients with advanced metastatic breast cancer and lesions refractory to standard chemotherapyday was proposed and studied in detail by Perroud *et al.* [29, 30]. They showed that this scheme was beneficial to almost half of the enrolled patients. The overall rate of clinical benefit was 46% with a median time to progression of 14 weeks with only mild gastric toxicity of grade 1 or hematologic toxicity of grade 1 or 2 reported [29]. They also revealed that this drug combination had an antiangiogenic effect [30].

Overexpression of COX-2 can result in poor outcome and resistance to platinum-based chemotherapy in ovarian cancer [44]. To find a way to improve platinum sensitivity, 400 mg of celecoxib per day were administered orally to patients treated with intravenous carboplatin for recurrent ovarian cancer that had exhausted treatment. Such a combination showed a long-lasting clinical benefit with median progression-free survival and overall survival of 8 and 17 months, respectively. This therapy was well tolerated by patients and their quality of life was preserved. It was also revealed that the urinary level of PGE-M (11α-hydroxy-9,15-dioxo-2,3,4,5-tetranor-prostane-1,20-dionic acid), which is the major metabolite of prostaglandin E2, could serve as a biomarker for the qualification of patients for therapy based on COX-2 inhibition [31].

The efficacy of celecoxib in combination with zoledronic acid, hormone therapy, and / or radiation therapy was assessed in a long-term randomized control trial in men with locally advanced or metastatic prostate cancer [32]. Celecoxib was administered at a dose of 400 mg per day for a year, while 4 mg of zoledronic acid was administered in 3–4 weekly cycles for two years. The results of this trial did not show a significant survival advantage. Similar findings were reported in a study that evaluated the clinical efficacy of 200 mg of celecoxib administered orally twice a day in combination with 25 mg/m^2^ of docetaxel and 4 mg of zoledronic acid [33]. However, a study conducted in Korean patients with metastatic castration resistant prostate cancer showed that oral metronomic chemotherapy consisting of 50 mg of cyclophosphamide per day, 1 mg of dexamethasone per day and 200 mg of celecoxib administered twice a day could be effective, safe and well tolerated [34].

In a phase III double blind randomized clinical trial, the efficacy of a metronomic therapy of advanced non-small cell lung cancer (NSCLC) at stage IIIB-IV with celecoxib was compared with *placebo* [35]. The drug (400 mg twice a day) was combined with standard palliative first-line chemotherapy consisting of carboplatin or cisplatin and gemcitabine or vinorelbine. In total, more than 300 patients participated in this study (158 in the control group). The results did not demonstrate an overall survival benefit. The differences observed between the *placebo* group and the celecoxib-treated group in quality of life and pain control were slightly better for the latter, but the difference was not statistically significant [35]. Six years later, the results of another double-blind clinical trial were published [36]. In that case, the efficacy of celecoxib in combination with carboplatin and pemetrexed or carboplatin and gemcitabine was studied in non-squamous or squamous NSCLC, respectively. The findings were in line with those of the previous study, as there were no significant differences between the celecoxib-treated groups and the control group. However, the results of a meta-analysis, comparing celecoxib plus standard chemotherapy *versus* chemotherapy alone, showed that this selective COX-2 inhibitor could improve the overall response rate in patients with NSCLC [45].

In addition to metronomic therapy, celecoxib can also be an interesting option for chemoprevention to block, reverse, or delay the development of invasive colorectal cancer. This idea comes from the fact that the overexpression of COX-2 was found in tumors, adenomas, and inflamed colon mucosa, while it was absent in healthy tissues [46]. Thus, it was assumed that oral administration of selective COX-2 inhibitors could replace or delay prophylactic surgery and endoscopic surveillance in patients with persistent adenoma, polyps, or hereditary predispositions to develop colorectal cancer. A recent meta-analysis confirmed that celecoxib could significantly reduce the incidence of advanced colorectal adenocarcinomas, especially in patients with a low risk of cardiovascular events [47]. The authors concluded that the recommended drug dose should not be greater than 400 mg per day and, preferably, the drug should be administered once a day. At the same time, they drew attention to the need for long-term clinical studies to assess the efficacy and safety of this approach.

Interestingly, oral administration of celecoxib was also applied to treat a common skin disorder related to systemic chemotherapy that is known as hand-foot syndrome. Such patients suffer from burning pain, erythema, desquamation, or even skin ulceration and infection. In severe cases, hand-foot syndrome can lead to discontinuation of treatment. This adverse reaction often occurs after standard chemotherapy with capecitabine, 5-fluorouracil, PEGylated liposomal doxorubicin, sorafenib and sunitinib [48]. Its mechanism remained long unclear, but its inflammatory basis was taken from the beginning into consideration as one of the possible scenarios. For that reason, the efficacy of celecoxib in oral treatment of hand foot syndrome was first investigated in the clinic more than a decade ago [48]. The results of these studies revealed that celecoxib should be administered in the dose range of 200–400 mg twice a day, which in turn may be related to cardiovascular events, especially if patients were at the risk group or if cardio toxic drugs were used in chemotherapy. To overcome these problems, a recent pilot trial described by Shayeganmehr *et al.* [49] showed that hand-foot syndrome can be treated with a topical hydrogel loaded with 1% celecoxib. The matrix of this hydrogel was composed of carbomer (1%), absolute ethanol (33%), glycerin (15%) and PEG 400 (30%) dispersed in water. The hydrogel was applied to the skin twice daily for three weeks. The drug was said to penetrate the stratum corneum and was distributed in the epidermis and dermis. Importantly, most of the drug was retained in the epidermis, indicating that the systemic absorption of celecoxib was limited, as was the risk of systemic adverse effects. Furthermore, the combination of metabolomics with cell RNA sequencing allowed them to demonstrate that interleukins overexpression (IL-6 and IL-8) could be related to the hand-foot syndrome apart from the overexpression of COX-2. Therefore, the authors suggested that in addition to COX-2 inhibition, celecoxib was also able to control the level of these interleukins [49].

### Antimicrobial Activity

Repurposing approved drugs is an important strategy to accelerate the development of new antimicrobial therapies that could face the emergence of bacterial resistance. The first reports of antibacterial activity of celecoxib date back to 2009 when Chiu *et al.* [50] demonstrated that the drug inhibited the proliferation of an intracellular Gram-negative bacterium *Francisella tularensis.* This highly virulent pathogen causes tularemia zoonotic disease in humans that is currently considered a category A bioterrorism agent. Surprisingly, the antibacterial activity of celecoxib was much higher than that of rofecoxib, which is the strongest COX-2 inhibitor. Further studies of this research team also revealed that celecoxib shows modest antibacterial activity against methicillin-resistant *Staphylococcus aureus* (MRSA)*.*

Although in the other study where celecoxib was used alone, it did not show a bactericidal nor bacteriostatic effect [51], its combination with antibiotics reversed multidrug resistance in MRSA and improved *Mycobacterium smegmatis* and *Staphylococcus aureus* sensitivity to standard treatment. It was stated that celecoxib increased ampicillin uptake by *Staphylococcus aureus* by affecting the Na^+^/K^+^ ion transporter and altering membrane potential and permeability [52]. Importantly, the synergy of celecoxib was observed with regard to both topical and systemic antimicrobials, which may prolong their clinical efficacy in the therapy of resistant strains of bacteria. Recently, Thangamani *et al.* [53] have elucidated the mechanism by which celecoxib interacted with bacterial cells. They demonstrated that the drug inhibited RNA, DNA and protein synthesis in a dose-dependent way. Furthermore, the level of inflammatory cytokines, such as IL-6, TNF-α, IL-1β, and MCP-1, was significantly reduced after topical administration of the formulation loaded with 2% celecoxib. This may suggest that celecoxib topical treatment could prevent scar formation by accelerating wound healing, which is a common complication in bacterial skin infections caused by MRSA strains.

These favorable features of celecoxib encouraged the design of new structural analogues of this drug that show a stronger antimicrobial effect [54]*.* In recent years, the properties of AR-12 were studied in detail. This drug candidate (without COX-2 inhibition) was qualified for the phase I clinical trial due to its antitumor activity. AR-12 was found to be an ATP-competitive, time-dependent inhibitor of yeast acetyl coenzyme A synthetase, which governs the growth of microorganisms [55]. Inhibition of this enzyme resulted in autophagy and loss of cellular integrity. As a consequence, AR-12 was declared to be fungicidal at concentrations similar to those achieved in human plasma [55, 56]. It also showed synergy with fluconazole in dealing with cryptococcal meningitis. Finally, this derivative showed a wide spectrum of antimicrobial activity, as it was also effective against *Candida albicans* biofilms, yeast, molds and dimorphic fungi, including resistant species [56]. In addition, AR-12 was able to potentiate the antibacterial activity of polymyxins (colistin and polymyxin B) in the treatment of multidrug-resistant bacteria, which are considered the last line of defense against *Klebsiella pneumoniae*, *Acinetobacter baumannii*, *Enterobacter* sp., *Escherichia coli,* and *Pseudomonas aeruginosa*.

In 2015, a plasmid-mediated polymyxin resistance mechanism was reported in *Enterobacteriaceae,* which was a threat to the polymyxin therapy. To address this problem, the idea of using a small molecule adjuvant was proposed and the suitability of AR-12 for this purpose was also investigated. It was confirmed that the MIC (minimum inhibitory concentration) of polymyxins and the number of colony formation units can be reduced if combined with AR-12. The results of this study also suggested that AR-12 may also facilitate antibiotic uptake in strains of *A. baumannii* and *K. pneumoniae,* increasing their antibacterial activity [57]. Other celecoxib derivatives, named Cpd36 and Cpd46, were found to be effective in the treatment of antibiotic-resistant *Staphylococcus aureus* infections [58]. These compounds targeted the YidC2 translocase membrane protein in bacteria, which led to a reduction in ATP production, and as a result, blocked the cell growth. Importantly, Cpd46 was noted to also be used to eradicate MRSA persisters and biofilms [58].

Coronavirus disease 2019 (COVID-19), caused by severe acute respiratory coronavirus-2 (SARS-CoV-2) that is the RNA betacoronavirus of the *Coronaviridae* family, has been one of the most challenging infections for the last three years. COVID-19 is recognized as a systematic hyper-inflammatory disease with reported high levels of prostaglandin E2 (PGE2) and cytokines in serum. Such a high PGE2 level could be the aftermath of the stimulation of COX-2 activity by the virus. A short-term celecoxib treatment was studied in patients with mild to moderate COVID-19 infection (Table I). Participants were divided into three groups, taking a recommended routine treatment of the guideline and 200 mg or 400 mg of celecoxib per day *vs.* 500 mg of naproxen per day. Routine treatment included isolation, antiviral medication, antibiotics, glucocorticoids, oxygen therapy, and assisted breathing, if necessary. The group taking 400 mg of celecoxib daily showed a higher oxygen saturation level compared to the other groups. Therefore, celecoxib has been reported to be a suitable candidate for the treatment of COVID-19 infection [37]. Another prospective clinical trial confirmed these findings. Adjuvantive administration of celecoxib at doses of 200–400 mg per day, in combination with the above-mentioned routine treatment, promoted recovery from ordinary to severe COVID-19 and significantly reduced mortality in elderly patients [38].

The Venezuelan equine encephalitis virus (VEEV) is another RNA virus that could spread throughout the world, causing epidemics. It is an alphavirus of the *Togaviridae* family. The first symptoms of VEEV infection are high fever, headache, and general malaise [59]. Inflammation related to this infection may result in neurological complications with damage to the blood–brain barrier, because, in response to infection, the macrophages in the central nervous system release pro-inflammatory cytokines. Thus, the efficacy of three anti-inflammatory drugs with different mechanism of action, i.e., celecoxib, rolipram and tofacitinib, was compared in the treatment of VEEV infection. Among them, celecoxib was found to be the strongest anti-VEEV molecule, inhibiting virus replication up to six hours after exposure. Furthermore, the levels of IL-1_A_, IL-17_F_ and TNFα cytokine genes were also reduced. These results showed the potential to use celecoxib as a countermeasure strategy to slow the development of VEEV infection [60].

### Mental Disorders

Multiple studies investigated the functionality of celecoxib in the treatment of mental disorders, such as depression. Understanding the relationship between inflammation and depression has recently gained more and more attention. Balance between inflammatory cytokines: IL-1, IL-6, and TNFα was reported to be disturbed in patients with a major depressive disorder. These cytokines influence prostaglandin synthesis and activate the hypothalamic–pituitary–adrenal axis, affecting adrenergic and serotonergic neurons. Therefore, COX-2 inhibitors blocking PG synthesis could reduce inflammatory processes and improve depression symptoms [61]. The results of the meta-analysis carried out by Wang *et al.* [62] showed that 400 mg of celecoxib per day may have a beneficial impact on the treatment of depression. The drug was co-administered with sertraline (50–200 mg daily), fluoxetine (40 mg daily), escitalopram (20 mg daily) or reboxetine (4–10 mg daily). However, the authors stressed that not all treated patients had an abnormally high level of pro-inflammatory cytokines. In these patients, celecoxib probably would not improve depression [62].

Inflammation is supposed to also play a role in the etiopathogenesis of bipolar disorder [BD] [63]. Drugs currently used in the management of BD, such as lithium, carbamazepine, valproic acid, lamotrigine, and olanzapine, reduce the turnover of arachidonic acid (AA). In response to the agonism of the N-methyl-D aspartic acid (NMDA), D2, or 5-HT2A/2C receptor, they decrease brain AA signals. In turn, direct COX inhibitors interfere with AA-mediated neurotransmission, and therefore; could be useful as an adjunctive therapy of BD [64]. Furthermore, the review by Bartoli *et al.* [65] concluded that it was more likely to achieve clinical remission after 6–8 weeks of celecoxib adjuvant administration compared to *placebo*.

In addition to bipolar disorder, celecoxib was also co-administered in the context of adjunctive therapy in schizophrenia. This disorder is associated with altered levels of pro- and anti-inflammatory cytokines, and abnormal cytokine profiles depending on the disease phase [66]. Celecoxib was effective, especially in the early stages and in acute schizophrenia. In more chronic cases, benefits were not reported. When celecoxib was administered at a dose of 400 mg per day in combination with risperidone or amisulpride, the clinical outcome was significantly better compared to *placebo* [67].

Similar findings have been reported in studies focused on the impact of adjuvant celecoxib therapy may have on obsessive–compulsive disorder (OCD). The most investigated mechanism of this disease is the dysregulation of serotonergic systems, but the influence of inflammation has been extensively explored recently [39]. The combination of 400 mg of celecoxib per day with 20 mg of fluoxetine per day showed a greater improvement in obsessive and compulsive symptoms compared to fluoxetine administered alone [40]. Then, co-administration of 400 mg of celecoxib per day as an adjuvant to fluvoxamine also proved to be significantly superior to fluvoxamine monotherapy [41]. More studies are planned to establish the impact of celecoxib on childhood OCD, because the behavioral effects of COX inhibitors may not only be related to the anti-inflammatory path, but could also result from direct effects that celecoxib may have on synaptic transmission and neuronal function [68].

## Design of Modern Dosage Forms with Celecoxib

### Approaches to Enhance Oral Bioavailability of Celecoxib

New clinical indications may require the development of dosage forms that are adjusted to patients’ needs. Taking into account the unfavorable physicochemical properties of celecoxib, several attempts have been made to improve its aqueous solubility and dissolution rate with the aim of achieving a faster onset of action, minimizing the variability in oral absorption, and finally reducing the drug dose by increasing bioavailability. To face this challenge, multiple micro and nanoparticle-based delivery systems have been proposed so far [69]. Their development would not have been possible without new carriers and their combination with celecoxib using modern technologies. For that purpose, the functionality of multiple mesoporous carriers has been investigated throughout the recent years (Table II).

**Table II.** Mesoporous Carriers Used to Enhance Release Rate and Bioavailability of Celecoxib After Oral Administration

Kind of porous carrier	Pore size [nm]	Pore shape	Specific surface area [m^2^/g]	Drug loading method	Drug load [%]	ICH class of organic solvents	Results	References
Mesoporous carbon	n.a	Spherical	601	Solvent evaporation: ethanolic solution of celecoxib combined with carrier, ethanol evaporated at RT or melting: celecoxib melt combined with carrier, reheated again (165 °C), finally cooled down in liquid nitrogen	19–37	3	After solvent evaporation reduced celecoxib crystallinity, controlled release rate, After melting amorphous drug release with burst effect; Both drug incorporation methods ensured better celecoxib absorption in Caco-2 cells and oral bioavailability in rats was 1.59 times higher than that of commercial product	[70]
Monocellular carbon foam	25	Spherical	1257	Solvent evaporation: ethanolic solution of celecoxib combined with carrier, ethanol evaporated at RT	43	3	Celecoxib in amorphous form, ninefold increase in celecoxib solubility, immediate release of celecoxib, oral bioavailability in rat model was 1.72-fold higher than that of Celebrex®	[71]
Magnesium carbonate or Magnesium aluminometasilicate	1.4–6 10–20	n.a	332 436	Ethanolic solution of celecoxib combined with carrier, ethanol evaporated using rotary evaporator (75 °C)	33	3	Stabilization of amorphous celecoxib, improved dissolution kinetics, gradual increase in drug transfer over Caco-2 cell membrane Burst release of amorphous celecoxib, high supersaturation level followed by rapid recrystallization, initially high flux over Caco-2 cell membrane, after 15 min flux decreases	[72]
Calcium carbonate (Vaterite)	3–20	Bottleneck	57	Celecoxib dissolved in methanol, then loaded inside mesopores upon synthesis of vaterite from Ca(OH)_2_	25	2	Amorphous celecoxib stabilized inside mesopores; optimum load of celecoxib 15–25 vol.% apparent solubility 5- to sixfold higher than that of crystalline celecoxib	[73]
Porous silicon	17–58	n.a	77–243	Wetness impregnation of silicon particles with acetone solution of celecoxib	5–36	3	The lower the degree of celecoxib, the slower drug recrystallization on the surface, the higher release rate, high permeability through the Caco-2 monolayer, high oral bioavailability in rats	[74]
Mesoporous silica with core–shell structure	n.a	n.a	891	Methanolic celecoxib solution combined with the carrier, methanol evaporated at RT	19	2	Amorphous celecoxib of oral bioavailability (rat model) 9.9-fold and 1.89-fold higher than that of crystalline celecoxib or commercial drug product respectively	[75]

*n.a. *not available, *RT *room temperature

Among them, there are mesoporous grades of carbon [70, 71], calcium or magnesium carbonate [73], silica [75], silicates [72] or silicon [74]. Excellent stability and a large specific surface area, together with tunable morphology, pore size, and narrow pore size distribution, provide the opportunity to uniformly adsorb the drug on the surface of such a carrier and stabilize its amorphous form, whose release rate, permeability, and oral bioavailability were found to be much better than its crude crystalline counterpart [70–75]. Despite the solubility gain offered by the amorphous form of celecoxib, the stability of supersaturation created in the gastrointestinal tract is often too short to effectively enhance its oral bioavailability. Therefore, the development of lipid-based formulations that could maintain the drug dissolved upon digestion of lipids is an interesting option for oral delivery of lipophilic drugs. Chavan *et al.* [76] compared the functionality of four grades of micro or mesoporous silicon dioxide for the manufacturing of a supersaturable solid self-emulsifying drug delivery system loaded with celecoxib. The drug was dissolved in a liquid self-emulsifying system composed of an oil (Capryol 90), a surfactant (Tween 20), a cosurfactant (Transcutol HP), and a precipitation inhibitor (Soluplus). This liquid formulation was adsorbed onto porous silicon dioxide to finally form a free-flowing powder that could be filled in capsules. Since the specific surface area and hydrophilic/hydrophobic characteristics of the solid carriers differed (110–300 m^2^/g), the drug loading, its release rate and oral bioavailability differed as well. The best performance was stated for hydrophilic mesoporous silicon dioxide (Sylysia 350 fcp) with the highest specific surface area (300 m^2^/g). Furthermore, the application of the precipitation inhibitor (Soluplus) to stabilize the supersaturation formed inside the lumen was reported to maintain the concentration of celecoxib within the therapeutic range for 24 h (rat model). These results showed that proper selection of the porous carrier and the precipitation inhibitor may be helpful in adjusting the delivery of celecoxib to the patient’s needs. Importantly, these silica-lipid hybrid formulations can be prepared on a large scale using spray drying [77] or freeze drying technology [78].

Despite the benefits of porous carriers, there are also important drawbacks of this technological approach such as a low drug load (about 30%), the risk of drug deposition on the outer surface of the carrier instead of inside the pores, or poor compressibility of the obtained powder. In solvent methods, the drug load can be increased while using nonpolar organic solvents, i.e. hexane [79]. Moreover, the application of incipient wetness impregnation when a concentrated drug solution in an organic solvent is prepared but its volume is precisely adjusted to the total volume of the pores in the carrier, instead of classical adsorption or evaporation methods where relatively high volumes of the drug solution in organic solvents are applied, can also be helpful to increase the drug load inside the pores [79]. However, if class 2 organic solvents are applied, the level of their residuals should be controlled according to the current ICH requirements (Table II). In this context, the application of supercritical fluid technology, comilling, or the development of alternative solvent-free methods is worth investigating.

### Design of Long-Acting Injectables for Celecoxib Delivery

Since there is a risk of severe side effects related to systemic administration of celecoxib and corticosteroids upon chronic anti-inflammatory treatment, Yang *et al.* [80] developed microspheres loaded with celecoxib and triamcinolone acetonide using poly(lactic-co-glycolic acid) coupled with polythioester (PLGA-PTE) as a matrix-forming polymer (Table III). This formulation was destined for intraarticular administration of both drugs in order to control pain associated to degenerative musculoskeletal diseases. The results of *in vitro* studies carried out in TNF-stimulated chondrocyte cultures stimulated with TNFα revealed that the drugs released from the microspheres inhibited the production of PGE2. As their matrix ensured prolonged release, the anti-inflammatory effect lasted three weeks after a single administration, which can limit the risk of infections often related to frequent injections.

**Table III.** Examples of Nano and Microparticles Loaded with Celecoxib (CXB) as Approaches to Develop Long-Acting Injectables

Route of administration	Drug form (carrier/vehicle/stabilizer)	Particle size [µm]	Technology	Type of test	Results	References
Intraarticular	Microspheres (PLGA-PTE)	40–55	Double emulsion	*In vitro* TNF-*α* stimulated osteoarthritic human chondrocytes	CXB-loaded microspheres inhibited PGE2 production for up to 21 days	[80]
Intramuscular	Nanocrystals (PVP K17 or TPGS or poloxamer 188)	2.1–2.4	Wet milling	*In vivo* rat model	Analgesic effect on acute pain for 72 h after single administration	[81]
Intraocular	Solution in DMSO	n.a	n.a	*In vivo* rabbit model	Detectable levels of CXB in the retina/choroid after 8 weeks	[82]
Intraocular	Nanoparticles (poly(ortho ester), poloxamer 188)	0.151–0.164	Double emulsion solvent diffusion technique	*In vitro* Müller cells	Controlled release of CXB for 14 weeks	[83]

*DMSO *dimethylsulfoxide, *n.a. *not applicable, *PVP *povidone, *TNF-α *tumor necrosis factor alpha, *TPGS *D-α-tocopheryl polyethylene glycol 1000 succinate

Recently, long-acting intramuscular injections loaded with celecoxib nanocrystals have been designed for the treatment of acute postoperative pain [81]. They were prepared using wet milling technology. The impact of various stabilizers on *in vitro* drug release, pharmacokinetics, pharmacodynamics, and safety of the final formulation was studied in rodents. Among them, there was a polymer, polyvinylpyrrolidone K17, and surfactants poloxamer 188 or D-α-tocopherol polyethylene glycol 1000 succinate. The first stabilizer was found to be the least favorable, whereas the latter ensured the highest release rate, the highest maximal concentration in rat plasma, and the longest drug elimination. Although the kind of stabilizer determined the drug performance *in vitro* and its pharmacokinetics, the analgesic effect was always observed. Furthermore, these intramuscular injections were biocompatible as neither systemic toxicity nor local inflammatory reactions were reported.

Due to the fact that celecoxib can reduce the expression of retinal VEGF [84], some research studies focused on the development of long-acting ocular injections (83, 84]. Retinal VEGF plays a role in the pathophysiology of diabetic retinopathy and age-related macular degradation (AMD), which are common causes of blindness [85]. Topical administration of celecoxib in the form of eye drops is therapeutically ineffective by cause of considerable drug losses during administration. On the other hand, when celecoxib is administered orally, high doses of the drug are necessary to obtain its therapeutic concentration in the retina. Upon chronic treatment, it may result in severe adverse effects. Therefore, transscleral injections have been proposed as an alternative to oral treatment [86].

Kim *et al.* [82] examined the efficacy and safety of the celecoxib solution in dimethylsulfoxide injected intravitreally in rabbits. The formulation was nontoxic and, in general, well-tolerated. However, cataract formation was observed when the celecoxib dose was higher than 6 mg, which could be related to the rapid drug precipitation after injection. Importantly, the study showed that a single injection of celecoxib ensured sustained drug delivery for up to 8 weeks, which was accompanied by a significant reduction in PGE2 level. In turn, Palamoor and Jablonski [83] described the manufacturing method and properties of celecoxib-loaded poly(ortho ester) nanoparticles with lactic acid designed for the sake of diabetic retinopathy and AMD treatment. A double emulsion ([o/w]/w) solvent diffusion technique was applied to prepare the hydrophobic formulation of the prolonged celecoxib release, which relied on surface erosion. The advantages of these nanoparticles were high drug encapsulation and low cytotoxicity.

### Modern Strategies for Topical Celecoxib Delivery

The main advantage of topical application is the opportunity to overcome the risk of cardiovascular side effects that could be associated with the systemic administration of the drug. Furthermore, the poor solubility in water and, consequently, the limited bioavailability of celecoxib after oral administration encourages the development of formulations that would enable its topical administration (Table IV). The growing interest in nanostructural lipid carriers (NLCs) is due to the fact that they can serve as a universal delivery platform for insoluble drugs, enabling their successful administration through various routes [87–89]. When applied to the skin, NLCs are not able to penetrate the stratum corneum. They usually form an occlusive layer from which the drug is released in a sustained way. That was the case for the gel loaded with celecoxib [90]. The drug permeability of this formulation was faster and lasted longer (up to 24 h) than that of a micellar gel. Therefore, NLC encapsulation inside cell penetrating cationic peptides that penetrate the cells, such as polyarginine-11, was proposed as a way to increase celecoxib delivery to deep layers of the skin [91]. These findings can be translated into modern formulations destined for the therapy of skin inflammatory diseases.

**Table IV.** Technological Approaches to Effectively Treat Inflammation Via Topical Administration of Celecoxib

Kind of celecoxib carrier	Technology	Excipients	Results	Particle size/Vesicle size [nm]	References
Nanostructured lipid carriers in gel matrix	Microemulsion template technique	Emulsynt (glyceryl dilaurate), Capmul MCM, Cremophor RH40, Transcutol gelling agent: Carbopol (Ultrez 10)	Faster onset of action Prolonged drug release as compared to micellar gel	160, 185 (before and after gelling respectively) PDI = 0.624	[90]
	Hot-melt high pressure homogenization	Compritol 888 ATO, Miglyol 812, DOGS-NTA-Ni, Tween 80; surface of NLC covered with histidine tagged peptides	Enhanced skin penetration of celecoxib	134 ± 11, 145 ± 15 (before and after surface modification respectively) PDI = 0.15	[91]
Nanoemulsified systems containing lamellar phases	Low-energy simple emulsification	Shea butter, sunflower oil, oleic acid, soybean phosphatidylcholine	Better penetration of drugs without irritation Increased cytotoxicity in breast cancer cells Co-delivery of celecoxib and endoxifen possible with synergic effect	291.5–453.4 PDI: 0.18–0.43	[92]
Spanlastics	Spraying technique/modified injection method	Span 60, Tween 80/Brij 35, absolute ethanol, 9% (w/v) sucrose solution, Gelling agent: Carbopol 934	Nanovesicles for arthritis therapy Celecoxib-loaded spanlastic gel showed better reduction of edema arthritis markers in rat model than niosomes	113–154 PDI: 0.126–0.321	[20]
Niosomes		Span 60, cholesterol, ethanol, sucrose solution Gelling agent: Carbopol 934		165–327 PDI: 0.213–0.245	

DOGS-NTA-Ni—1,2-dioleoyl-sn- glycero-3-[(N-(5-amino-1-carboxypentyl) imidodiacetic acid) succinyl nickel salt], *PDI *polydispersity index

In their recent research study, Mojeiko *et al.* (92) have described the properties of a nanoemulsion loaded with celecoxib and endoxifen (a non-steroidal selective estrogen receptor modulator) stabilized by a viscous bilayer lamellar phase formed by self-aggregated soybean phosphatidylcholine (liquid crystals) in an aqueous environment. The aim of this approach was to promote the simultaneous percutaneous penetration of these drugs, and in that way, provide an alternative to the oral chemoprevention in patients with breast cancer. Since they differed in aqueous solubility, poorly soluble celecoxib (1%) was incorporated into the lipid phase, while endoxifen (1%) was dissolved in water. Sunflower oil and shea butter were used as the lipid phase. In addition, caprylic acid or oleic acid were used as penetration enhancers. The results of the cytotoxicity studies carried out on MCF-7 breast cancer tumor cells showed that when this multidrug nanoemulsion was applied, the viability of this cell line decreased compared to single component nanoemulsions or conventional solutions. However, recent results from a randomized control trial have provided evidence that dermal application of the *Z* form of endoxifen (10 or 20 mg per day) in a liquid formulation was associated with severe skin rashes, which were the reason for discontinuation of therapy after just a few weeks [93]. Therefore, it was concluded that despite the important benefits of this approach, such as the absence of systemic adverse reactions and a significant reduction in mammographic breast density, the severity of these dermal reactions excludes the topical use of *Z*-endoxifen.

The development of celecoxib-loaded spanlastics is an interesting idea for topical/transdermal drug delivery, which has recently been proposed as a modern strategy for therapy of arthritis [20]. Spanlastics are a kind of modified unilamellar or multilamellar niosomes that are devoid of cholesterol-related rigidity and instead are highly elastic. Similarly to niosomes, they are made of a nonionic surfactant (e.g. spans—sorbitan esters) or their blends, but in addition, they also contain so called ‘an edge activator’, which is usually a wetting agent, reducing the interfacial tension (e.g., Tween 80, polyvinyl alcohol, Brij 35) and destabilizing the lipid bilayer, making spanlastics flexible. Thus, these spherical nanostructures (180–450 nm) show a unique ability to squeeze themselves not only through the skin pores but also through the intracellular spaces of the main skin barrier, the stratum corneum*.* As a result, a high drug load encapsulated within these nanovesicles can be transported to deep skin layers, making both transdermal delivery and sustained release of the drug possible [94]. Alaaeldin *et al.* [20] examined *in vivo* (rat model) the properties of celecoxib spanlastics incorporated into the carbomer hydrogel in comparison to a formulation loaded with niosomes or unprocessed drug. They showed that spanlastics could enhance the clinical performance of celecoxib administered in the form of conventional semisolid medications or niosomes, as the level of inflammatory markers and edema was clearly reduced.

## Conclusions

This review provides evidence that selective inhibition of COX-2 opens several pathways for new therapeutic targets, because inflammation is the source of multiple diseases. The potential activity of celecoxib as adjuvant in metronomic chemotherapy as well as in the treatment of cutaneous adverse effects of conventional chemotherapeutic schemes, such as hand-foot syndrome, is of particular interest. Furthermore, in the era of the growing threat of the outbreak of another pandemic related to increased drug resistance, there is a need for new therapeutic protocols that could enhance the activity of conventional antimicrobials or that could offer antimicrobial activity by themselves. The same is true for the treatment of depression in which drug resistance is not uncommon. However, these advances in pharmacotherapy are impossible without the design of suitable dosage forms enabling, e.g., topical administration of celecoxib or ensuring its high bioavailability or controlled release. The review of research studies on technological approaches to develop new formulations loaded with celecoxib confirms that despite the unfavorable physicochemical properties of this drug, it is possible to manufacture modern dosage forms for its systemic or localized delivery.
